# Identification and characterization of the glutamine synthetase gene family in oat (*Avena sativa* L.) and the role of *AsGS2-2C* under drought stress

**DOI:** 10.3389/fpls.2025.1719654

**Published:** 2025-12-01

**Authors:** Mei Yang, Shanshui Zheng, Jingya Li, Shilun Tong, Mingzhi Zhang, Guowen Cui, Chenghao Che, Wenwen Zhang, Yaqian Wang, Taiming Zhang, Jian Teng, Bing Li, Hang Yin

**Affiliations:** College of Animal Science and Technology, Northeast Agricultural University, Harbin, China

**Keywords:** *Avena sativa* L., glutamine synthetase gene, drought stress, stress response mechanisms, genome-wide

## Abstract

Drought is a critical limiting factor for crop yield, posing a substantial threat to global food security and negatively influencing plant growth and development. As a crop used as both a grain and forage, the yield of oat *(Avena sativa)* is significantly affected by drought. Glutamine synthetase *(GS)* is a crucial enzyme in plant nitrogen metabolism and plays an essential role in nitrogen utilization, growth regulation, and yield formation. We used bioinformatics analysis to identify the *GS* gene family in oats, and employed molecular biology, genetics and plant physiology methods to investigate the drought-resistant function of *AsGS2-2C*. In this study, 11 oat *AsGS* genes were identified, and the gene family and expression patterns were analyzed. Our findings revealed that a majority of *AsGS* genes were upregulated under drought and salt stress, whereas they were downregulated in response to cold stress and abscisic acid treatment. Cloning and functional analysis of the *AsGS2-2C* gene revealed that transgenic tobacco overexpressing *AsGS2-2C* presented increased tolerance to drought stress in phenotype. Physiological analyses revealed an increase in antioxidant enzyme activities and a reduction in membrane damage in AsGS2-2C-overexpressing plants. Under drought conditions, the expression of stress-responsive genes (*Cu/Zn-SOD, MnSOD, CBL1, GR1, GAPC, Gln1-5*, and *BI-1*) was significantly elevated in *AsGS2-2C* transgenic tobacco. Interestingly, *ACR11, GLU, ERD10B, Hxk3* and *Ltp1* exhibited initial upregulation followed by subsequent downregulation. These findings provide valuable insights into the molecular mechanisms underlying drought tolerance mediated by *AsGS2-2C* in oat, offering potential targets for crop improvement against drought.

## 1 Introduction

Drought is a significant stressor that impedes crop growth and development, leading to reductions in yield and quality on a global scale. In China, the incidence of drought is increasing annually, resulting in direct economic losses and posing substantial threats to agricultural production, food security, and ecological environment stability (Mao et al., 2018). Drought induces a series of alterations in plant morphological, physiological, and biochemical characteristics, including wilting, stomatal closure, decreased chlorophyll content, and diminished photosynthesis and transpiration rates. It also leads to an increase in reactive oxygen species (ROS) levels and enhanced membrane lipid peroxidation. In oat, drought stress causes significant damage to chloroplasts and mitochondria, causing the degradation of grana and matrix thylakoids within chloroplasts. The PSII reaction centers are adversely affected, and the leaf fluorescence parameters are notably decreased (Zhang et al., 2017a; Yang et al., 2021). Nevertheless, plants have gradually developed antioxidant defense systems to mitigate the effects of environmental stresses. Drought stress markedly increases the levels of total phenols, soluble proteins, hydrogen peroxide, ascorbic acid, betaine, enzymatic activity, and leaf epidermal thickness in oat leaves, which enables the plant to resist drought (Shehzadi et al., 2019). Under conditions of severe drought stress, oat cultivars with greater drought tolerance present reduced membrane damage, more robust osmotic regulation, and elevated abscisic acid contents (Wang et al., 2017).

Glutamine synthetase (GS) is a pivotal enzyme in the nitrogen metabolism pathway of plants and is characterized by its high affinity for ammonium nitrogen (NH_4_^+^). In the presence of ATP, GS facilitates the synthesis of glutamine (Gln) from glutamic acid (Glu) and ammonium (NH_4_^+^). Under the catalytic action of glutamate synthase (GOGAT), Gln subsequently transfers its amide group to α-ketoglutarate, resulting in the formation of two molecules of Glu. Gln and Glu, which are organic compounds, are integral to the synthesis of biological macromolecules such as proteins and enzymes, and they play crucial roles in plant morphogenesis (Kusano et al., 2011; Gojon, 2017). GSs are typically categorized into two isoforms: cytoplasmic synthetases (GS1s) and plastid synthetases (GS2s). GS1s are predominantly localized in the cytoplasm and play a crucial role in the assimilation of primary ammonium (NH_4_^+^) as well as in the remobilization and transport of nitrogen, which includes the translocation of stored nitrogen during seed germination and the recycling of nitrogen during leaf senescence (Harrison et al., 2003). In contrast, GS2s are located within chloroplasts and mitochondria, where they participate in nitrite reduction and the assimilation of ammonia generated through photorespiration (Taira et al., 2004). GS1 and GS2 isoenzymes are generally found in the leaves of most plant species. However, GS2 isoenzymes are present exclusively in the leaves in soybean and spinach (Galant et al., 2011). To date, *GS* genes have been identified in several plant species, including six in Arabidopsis (Li et al., 2006), four in rice (Mondal et al., 2021), six in maize (Thomsen et al., 2014), and twelve in wheat (Wang et al., 2021).

GSs are integral to nitrogen utilization, growth and development, yield formation, and the response to abiotic stress in plants (Thomsen et al., 2014; Zhang et al., 2017b) (Silveira et al., 2003; Kusano et al., 2011). Overexpression of *GS* genes increases plant biomass and yield (Habash et al., 2001). Specifically, overexpression of the wheat *GS* gene was found to be associated with an increased number of grains per spike and elevated nitrogen content within the plant (Martin et al., 2006b; Habash et al., 2007; Gadaleta et al., 2013). Furthermore, GS2 and ferredoxin-dependent glutamate synthase (Fd-GOGAT) have been linked to the accumulation of grain protein (Habash et al., 2001). During the grain-filling stage, variations in GS activity influence the protein content and cooking quality of different rice varieties (Jin et al., 2007). Overexpression of the *GS2* gene has been shown to increase the growth rate of tobacco plants, decrease the NH_4_^+^ content in leaves, and increase the levels of glutamic acid and glutamine (Migge et al., 2000). Differential responses of GS to stress have been noted among various GS isoforms and tissue types. The *GhGLN1.1a* gene of cotton is upregulated under nitrogen treatment, and its inactivation negatively impacts nitrogen accumulation and nitrogen use efficiency (Li et al., 2024). Poplar plants overexpressing the *GS1a* gene present increased leaf area, dry weight, plant height, and nitrogen use efficiency (Man et al., 2005). Research indicates that plants utilize glutamate produced via the GS-GOGAT pathway as a substrate for synthesizing proline to maintain the antioxidant system and osmotic balance under stress conditions (Surender Reddy et al., 2015). Drought led to a reduction in proline accumulation and the expression levels of associated metabolic genes in the *Lotus corniculatus Ljgln2–2* mutant, decreasing stress tolerance (Díaz et al., 2010). There was a significant correlation between the expression levels of *OsGS2* and *OsGS1* and the drought tolerance of rice cultivars. The transcription of *OsGS1;1* was downregulated in the stems and roots of drought-sensitive cultivars, whereas that of *OsGS1;1* was upregulated in drought-tolerant cultivars (Singh and Ghosh, 2012). Additionally, the genes *TaGS1*, *TaGS2*, and *Ta4D.GSe* are crucial for enhancing drought resistance in wheat plants because they improve ROS scavenging and osmotic regulation (Yu et al., 2020; Yin et al., 2022a).

Oat (*Avena sativa* L.) is an annual crop cultivated globally for both grain and forage and holds substantial economic and social importance because of its rich nutritional profile and its impact on human health and livestock productivity (Rasane et al., 2015; Grundy et al., 2018; Hu and Sang, 2020; Kappachery et al., 2024). Oat is characterized by its high yield potential, adaptability to diverse soil conditions, and resilience to abiotic stress. However, this crop continues to experience heightened drought conditions and persistent challenges induced by climate change, which have significantly impaired oat production (Gong et al., 2022). While the role of *GSs* in plant stress responses has been documented (Yin et al., 2022c), the molecular mechanisms through which *GSs* confer drought tolerance in oat remain inadequately understood. This study sought to identify *AsGS* genes in oat and elucidate their function in enhancing drought tolerance. By investigating the regulatory mechanisms of *AsGSs* in the response to drought stress, this research aims to contribute to the development of new oat varieties with improved drought tolerance, thereby ensuring sustained yield and quality.

## 2 Materials and methods

### 2.1 Identification and characterization of oat *AsGSs*

We used the Docker tool, downloaded the gene family analysis mirror image (gene-family:2.0), and stored the docker analysis data compressed package in the corresponding path file to complete the setup of the analysis environment. The hidden Markov model (HMM) of the PF03951 domain was obtained from the Pfam 37.0 database (https://pfam.xfam.org/). The genome assembly files of oat were downloaded from the oat website (https://plants.ensembl.org/Avena_sativa_OT3098/Info/Index?sa=oans&_refluxos=a10). We copied the genomic files and HMM files to the path file. In the docker mirror image, we set the environment variables and then prepared the data such as the pep file, cds file and HMM file. We used the HMM files to search for protein files, and the members of *AsGSs* in the result files based on the E value (E value ≤ 1e^-5^). The protein sequences encoded by the six GSs of *Arabidopsis* were obtained from TAIR (https://www.arabidopsis.org/). The sequences of oat and *Arabidopsis* were both analyzed domains via the Conserved Domain Database (NCBI-CDD) (http://www.ncbi.nlm.nih.gov/cdd/). After confirming the consistency of the domains, chromosomal localization analysis is conducted on the screened sequences. Based on the position of the sequences on the chromosome, the starting codon within the sequences, and the length of the sequences at the same position, redundant sequences are removed. The *AsGS* family members was finally determined. The chromosomal location and sequence information of *AsGSs* in oat were obtained from an online database (https://plants.ensembl.org/Avena_sativa_OT3098/Info/Index?sa=oans&_refluxos=a10). The physicochemical properties of AsGSs, including amino acid length, molecular weight (MW) and isoelectric point (pI), were predicted via the ExPASy ProtParam website (www.expasy.org). Simultaneously, the subcellular locations of oat AsGS proteins were predicted by CELLO v.2.5 (http://cello.life.nctu.edu.tw/).

### 2.2 Phylogenetic analysis and structure and motif composition of oat AsGSs

The protein sequences of the 12 wheat GSs and the four rice GSs were obtained from the EnsemblPlants website (http://plants.ensembl.org/Triticum_aestivum/Info/Index; http://plants.ensembl.org/Oryza_sativa/Info/Index). The GS protein sequences of the four species were used to create multiple sequence alignments using ClustalW and to perform phylogenetic analysis using the maximum likelihood method with 1000 bootstrap replications via MEGA 11.0. Eleven AsGS genes were renamed according to their phylogenetic classification and position on the chromosome. Conserved domains and conserved motifs were analyzed via NCBI-CDD and the MEME website (http://meme-suite.org/), respectively, and were visualized via TBtools software (Chen et al., 2020).

### 2.3 Collinearity analysis of AsGSs and cis-element analysis of AsGS promoter sequences

The MCScanX tool was used to construct syntenic analysis maps for analyzing the syntenic relationships of *AsGS* genes via TBtools (Chen et al., 2020). Furthermore, the nonsynonymous (Ka) and synonymous (Ks) substitution rates of AsGSs and the Ks/Ka were calculated via TBtools on the basis of the synteny map of the *AsGS* genes (Chen et al., 2020). The promoter sequences were obtained from the oat genome file by extracting the 2,000 bp upstream of the AsGSs start codon (ATG). Cis-acting elements were analyzed using promoter sequences via the PlantCARE website (http://bioinformatics.psb.ugent.be/webtools/plantcare/html/).

### 2.4 Plant materials and treatments

In this study, oat (*Avena sativa* L. cv. Qingyin No. 1) was used as the plant material. Uniform and full oat seeds were selected for cultivation in vermiculite and watered with 1/2 Hoagland nutrient solution. All oat plants were grown in a greenhouse at 25/20°C and 55/70% relative humidity during the day/night under a 16-h light/8-h dark photoperiod. Three-week-old seedlings of exhibiting uniform growth were treated with 4°C, 15% PEG-6000, 150 mmol/L NaCl, 150 mmol/L NaHCO_3_ and 100 mmol/L ABA. The cold test was conducted in a temperature-controlled incubator using a gradient cooling method. It started at 24°C and decreased by 2°C every hour until it reached 4°C for the treatment. Drought, salt and alkali stress were all handled by soil irrigation, and the stress solutions were all prepared with 1/2 Hoagland nutrient solution. The ABA treatment was carried out by spraying, and the dosage was maintained until the droplets fell off the leaf surface. Root and shoot samples were collected at 0 h (CK) and at 3 h, 6 h, 12 h, 24 h, and 48 h after treatment for expression analysis of *AsGSs*. The roots, stems, leaves, leaf sheaths, inflorescences, young spikes, and spike stalks of boot stage oat plants were sampled for tissue-specific expression analysis of *AsGSs*. All the samples were set up with three biological replicates, snap-frozen at -80°C in liquid nitrogen, and stored at -80°C for subsequent analysis.

### 2.5 Quantitative real-time PCR analysis of *AsGSs* expression patterns

The expression of *AsGSs* in different tissues and in response to abiotic stress and ABA treatment were investigated via qRT–PCR. First, total RNA was isolated from oat samples using an Ultrapure RNA Kit (CWBIO, China). The RNA was tested using agarose gel electrophoresis. Then, first-strand complementary DNA (cDNA) was synthesized by reverse transcription with a HiScript^®^ II Q RT SuperMix for qPCR (+gDNA wiper) Kit (Vazyme, China) according to the manufacturer’s instructions. The quality of the cDNA was examined using the *UBC* gene as an internal reference for PCR and electrophoresis validation. Furthermore, the specific primers for *AsGSs* (**Supplementary Table S1**) were designed via the Integrated DNA Technologies website (https://sg.idtdna.com/page) for qRT–PCR, and the *UBC* gene (**Supplementary Table S1**) was used as an internal reference control. qRT–PCR was conducted in a Quantagene q225 real-time PCR apparatus (Novogene, China) using ChamQTM Universal SYBR^®^ qPCR Master Mix (Vazyme, China) according to the manufacturer’s instructions. Three independent biological replicates and three replicate reactions were included for each sample in this experiment. The relative expression levels of *AsGSs* were calculated using the comparative cycle threshold (Ct) method (2^−ΔΔCt^) (Livak and Schmittgen, 2001).

### 2.6 Isolation and cloning of the *AsGS2-2C* gene

The coding DNA sequence (CDS) of *AsGS2-2C* was amplified via PCR with 2×Phanta^®^ Max Master Mix (Vazyme, China) following the manufacturer’s instructions and degenerate primers (AsGS2-2C-F/AsGS2-2C-R, **Supplementary Table S1**) that were designed using Primer 5.0 software (Applied Biosystems). The PCR product of *AsGS2-2C* was inserted into the pCE2 TA/Blunt-Zero vector (Vazyme, China), then transferred into Escherichia coli *DH5α*, and sent to RuiBiotech Co. (Harbin, China) for sequencing. After successful sequencing, the obtained CDS of the *AsGS2-2C* gene was submitted to the NCBI database (GenBank accession No. PQ720318).

### 2.7 Subcellular localization of the AsGS2-2C protein

The pBWA(V)BS-AsGS2-2C-linker-OSGFP fusion plasmid was constructed by homologous recombination and seamless cloning of the AsGS2-2C linear product and pBWA(V) BS-OSGFP linear product. For this vector construction process, the 0-F/R and 1-F/R (**Supplementary Table S1**) primers were used for PCR, and the *BsaI* and *Eco31I* restriction enzymes were used to linearize the vector. The recombination products were subsequently transformed into Escherichia coli *DH5α* competent cells. The plasmids were subsequently extracted from positive clones using a FastPure Plasmid Mini Kit (Vazyme, China) and sequenced. The AsGS2-2C fusion protein was transformed into the leaf cells of *N. benthamiana* followed by 48 h of incubation at room temperature in the dark for transient expression (Yin et al., 2022b). Furthermore, a confocal laser scanning microscope (Leica TCS SP8, Germany) was used to identify the GFP fluorescence signal.

### 2.8 Plant transformation and generation of transgenic plants

The pBWA(V)BS-AsGS2-2C-linker-OSGFP fusion plasmid was used as an overexpression vector to be introduced into the Agrobacterium *EHA105*, which were used to infect wild-type (WT) tobacco (*Nicotiana tabacum* cv. k326) by the agroinfiltration method (Kaur et al., 2021), leading to the generation of AsGS2-2C-overexpressing (AsGS2-2C-OE) plants. After obtaining the transformed plants, first of all, we extracted DNA from each plant line separately. Using the extracted DNA as the substrate, we performed PCR amplification for the screening gene *Bar* (**Supplementary Table S1**). Based on the agarose gel electrophoresis, we initially determined the positive plant lines and obtained the T1 generation seeds. The T1 seeds were placed in the MS medium containing glufosinate to conduct germination screening. The T1 generation seedlings with better growth were selected for transplantation. Then, we extracted RNA from the T1 seedlings of each line and reverse-transcribed them into cDNA, respectively. Using the cDNA as the substrate, we determined the relative expression level of the *AsGS2-2C* gene through qRT–PCR with *NtActin* as a reference and tobacco without genetic transformation as the wild-type control. The T2 generation positive seeds were harvested. Based on their expression levels, we selected two strains with relatively high expression levels and continued to conduct glufosinate screening for T2 generation seeds. Those with better growth were homozygous lines, and the expression level of the *AsGS2-2C* gene was detected again. Homozygous lines transplanted were used in subsequent experiments to investigate the impact of *AsGS2-2C* regulation on plants under drought stress.

### 2.9 Physiological indices and gene expression level determination in homozygous tobacco lines subjected to drought stress

After a growth period of 3 weeks, the WT, OE#6, and OE#13 tobacco lines were subjected to drought via treatment with 20% PEG6000 nutrient solution, followed by observation of phenotypic changes. The plants subjected to 2 d and 6 d of stress were subsequently stored at −80 °C, after which the physiological indices and expression levels of relevant genes were measured. The malondialdehyde (MDA) content was quantified using the modified thiobarbituric acid (TBA) method (Puckette et al., 2007). Superoxide dismutase (SOD) activity was assessed using the nitro blue tetrazolium (NBT) method (Giannopolitis and Ries, 1977). Catalase (CAT) activity was assessed following Maehly’s procedures, and peroxidase (POD) activity was assessed using the guaiacol method (Polle et al., 1994). The proline content was quantified using the ninhydrin method (Bates et al., 1973), and the soluble sugar content was determined following the methods of Dreywood (Dreywood, 1946). The soluble protein content was assessed using the Bradford method (Bradford, 1976). All physiological indices were tested with kits from Suzhou Comin Biotechnology Co., Ltd.

Quantitative primers for 12 related genes (*GDH1*, *14-3-3*, *GR1*, *GAPC*, *CBL1*, *MnSOD*, *Cu/Zn-SOD*, *BI-1*, *Hxk3*, *Ltp1*, *ERD10B* and *Gln1-5*) across the transgenic lines were designed using Primer 5 (**Supplementary Table S1**), and their relative expression levels were evaluated via qRT–PCR with *NtActin* as the reference gene.

### 2.10 Statistical analyses

The experiments conducted in this study were replicated three times using the same methodology, and all the data are expressed as the means ± standard errors (SEs). Data processing was carried out using Excel 2019, and statistical analysis was performed using SPSS 22.0. Student’s t test and one-way analysis of variance were employed to determine significant differences for multiple comparisons according to the Duncan method. Graphs were generated using PowerPoint 2019, MEGA 11.0, GraphPad Prism 8.0, Origin 2018 and TBtools v2.096.

## 3 Results

### 3.1 Identification and characterization of *AsGSs* in oat

To identify and obtain the *AsGSs* in the oat genome, a global search of the oat genome was performed using the HMM profile of the HKD domain (PF03951) for sequence alignment, conserved structural domain analysis and other methods, identifying a total of 11 *AsGSs*, and these *AsGSs* were used for subsequent analyses (**Supplementary Table S2**). The 11 *AsGS* genes were renamed according to their phylogenetic classification and position on the chromosome, and the gene characteristics, chromosome locations, protein sequence lengths, molecular weights (MWs), isoelectric points (pIs), and subcellular locations are shown in **Supplementary Table S2**.

The lengths of the 11 AsGSs ranged from 326 to 431, their MWs ranged from 35.82 to 47.00 kDa, and their pIs ranged from 5.46 to 8.68. All the proteins appeared to be weakly acidic other than AsGS2-2D. The 11 *AsGS* genes were unevenly distributed on chromosomes 1 to 7 (**Supplementary Table S2**). The greatest number of genes (3) was found on chromosomes 2, 4 and 6, whereas only one gene was present on chromosomes 1 and 5. The subcellular localization prediction results revealed that most of the AsGSs were localized in the cytoplasm, whereas the others were localized in the extracellular space, chloroplasts and mitochondria.

### 3.2 Phylogenetic and protein sequence analysis of the *AsGS* gene family

**Figure 1:**
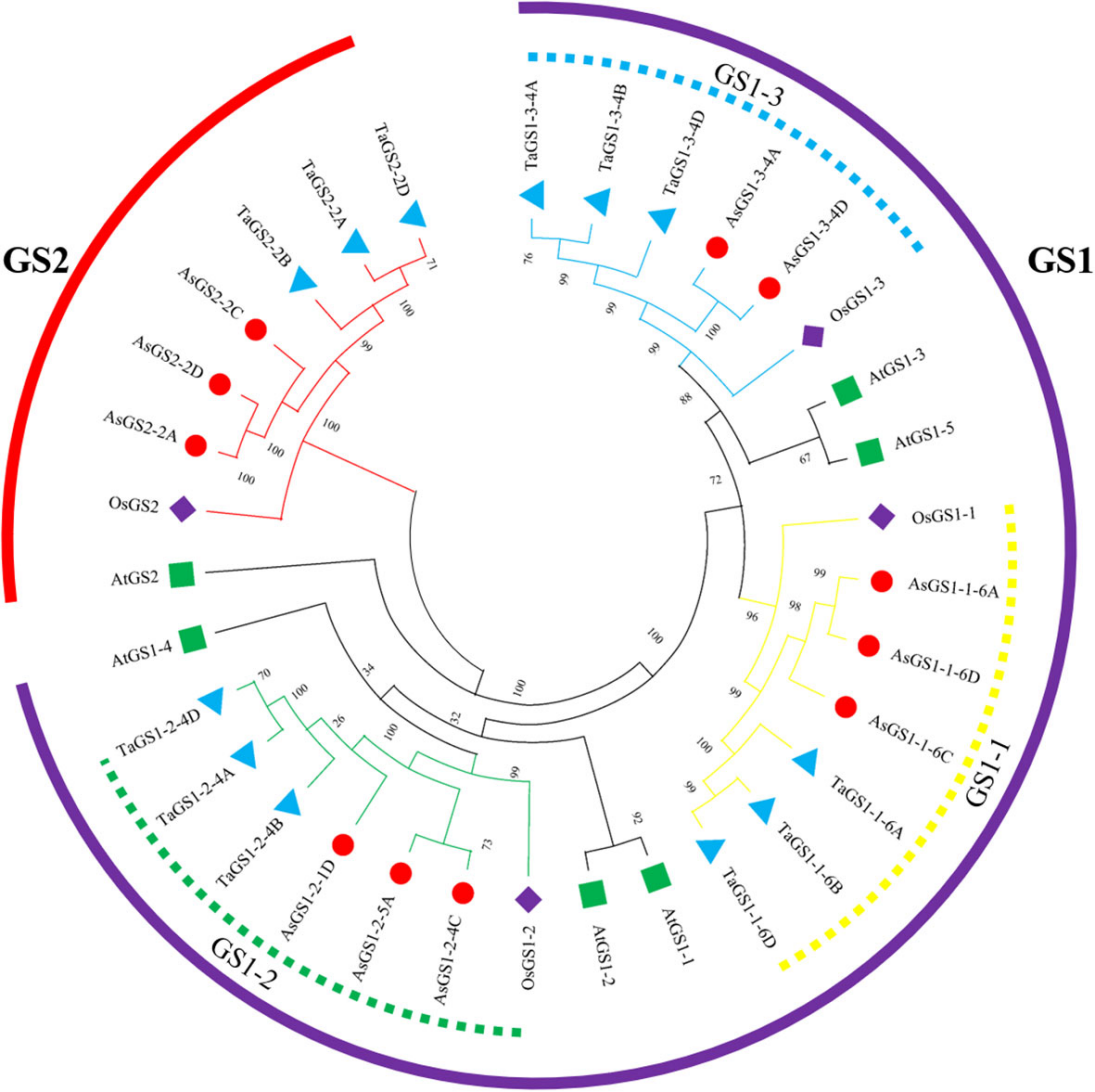
Phylogenetic analysis of GSs in *Avena sativa*, *Triticum aestivum*, *Oryza sativa* and *Arabidopsis*. The amino acid sequences of GS proteins from oat, rice, wheat and *Arabidopsis* were used for the phylogenetic analysis. The phylogenetic tree was constructed with MEGA 11.0 using the maximum likelihood method with 1000 bootstrap replicates. The different colors on the evolutionary tree branches represent different GS subfamilies, and the symbols ○, △, ◇ and □ represent GSs in oat, wheat, rice, and *Arabidopsis*, respectively.

To investigate the evolutionary relationships of *AsGS* gene family members, a phylogenetic tree was constructed using the amino acid sequences from *Arabidopsis*, rice, wheat and oat. Phylogenetic analysis revealed that the GSs of oat can be divided into GS1s and GS2s, and the GS1s can be divided into the GS1-1, GS1–2 and GS1–3 subgroups, among these, the GS1–1 and GS1–3 subgroups are the most closely related, which is in line with the results of previous studies in rice and wheat (**Figure 1**).

**Figure 2:**
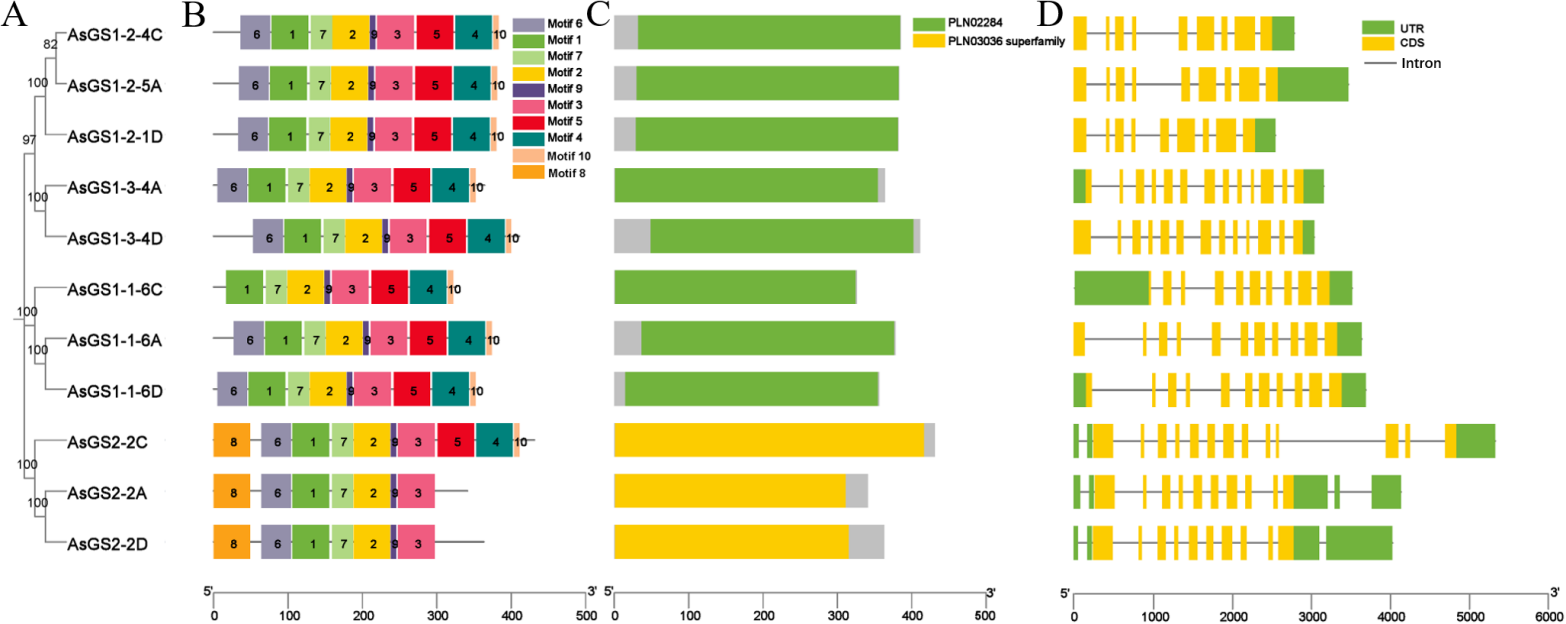
Sequence structure analysis of AsGSs in oat. **(A)** Phylogenetic analysis of the amino acid sequences of 11 AsGSs using MEGA 11.0. **(B)** Motif distribution of AsGS proteins. Different motifs (1–10) are indicated by different colors. The sequence logos and information for each motif are provided in **Supplementary Figure S2** and **Supplementary Table S3**. **(C)** Domain distribution of AsGS proteins. Different domains are indicated by different colors. **(D)** Exon–intron and CDS structures of AsGSs.

The diversity of the gene structures supported phylogenetic grouping to some extent (Wei et al., 2016). To better understand the sequence structure of *AsGSs* in oat, a phylogenetic evolutionary tree of 11 AsGSs was constructed (**Figure 2A**). Motif divergence was examined to gain more insight into the evolution of the 11 AsGSs (Seoighe and Gehring, 2004). A total of 10 motifs were predicted and used to analyze the features of the *GS* gene family in oat. The sequence information for each motif is provided in **Supplementary Figure S2** and **Supplementary Table S3**. As shown in **Figure 2B**, each AsGS contained between seven and nine motifs; some motifs were shared by all AsGS members, whereas others existed in only a few subgroups. For example, motifs 1, 2, 3, 7, and 9 were present in all members; motifs 4, 5, and 10 were present in all members except AsGS2-2A and AsGS2-2D; and motif 8 was detected in only AsGS2s and was identified as an important component of the PLN03036 domain. The CDD analysis revealed that the 11 AsGSs contained two domains between PLN02284 and PLN03036 (**Figure 2C**), and GS1-1, GS1–2 and GS1–3 had the same domain, which was different from that in GS2.

The exon–intron data of AsGSs revealed that the structures of the *AsGS* genes were different overall but were similar among the same isoforms (**Figure 2D**). Among the *AsGSs*, gene members belonging to the AsGS1–2 subgroup contained 8 introns, and those belonging to the AsGS1–3 subgroup contained 12 introns. In contrast, the number of introns in the AsGS1–1 and AsGS2 members ranged from 9 to 12. These results indicated that the *AsGSs* were conserved and specific, and the similar gene structures of different *AsGSs* in the same subfamily were consistent with their phylogenetic relationships (**Figure 2A**).

### 3.3 Collinearity and gene duplication analysis of *AsGSs*

**Figure 3:**
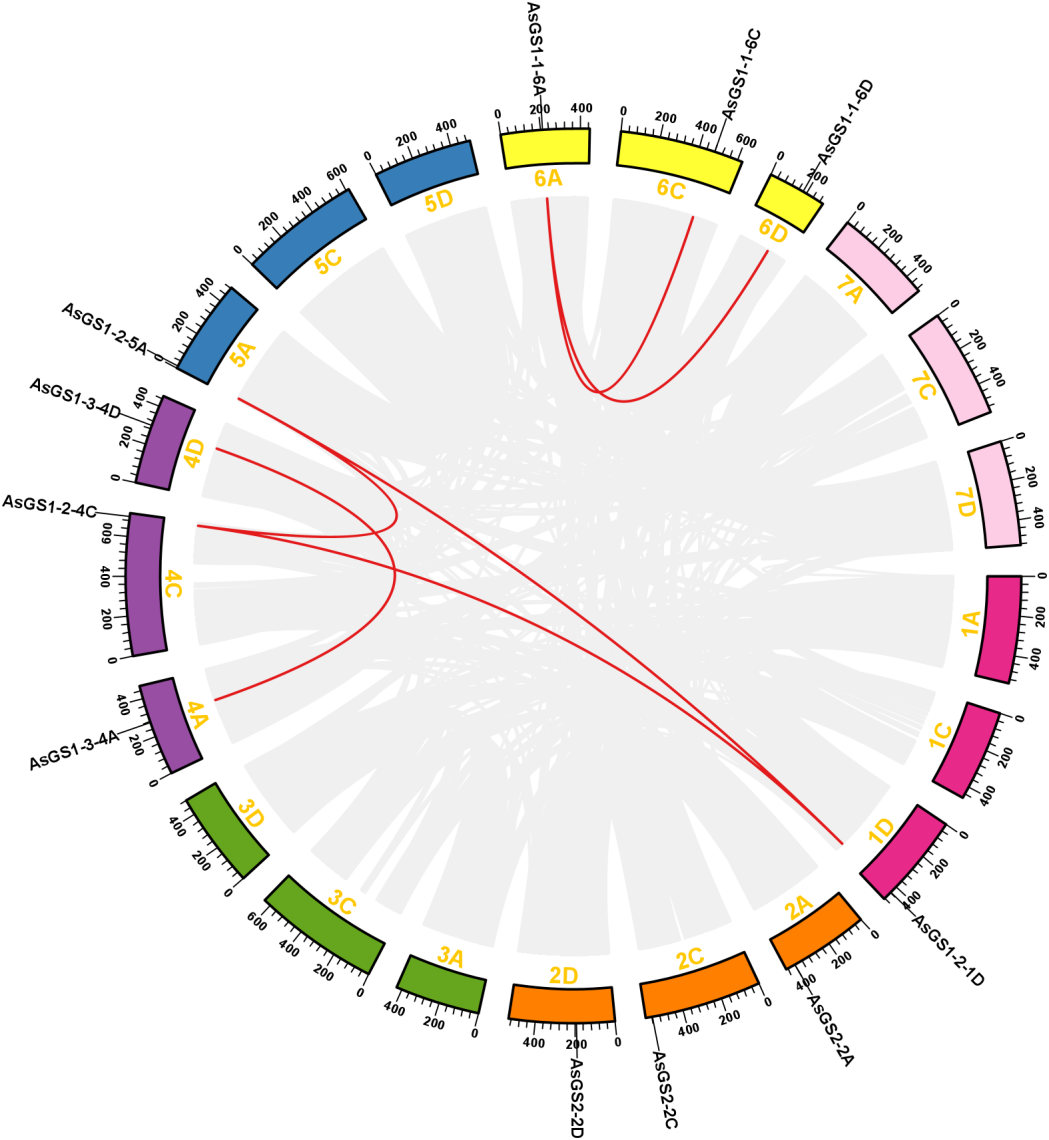
Gene duplication analysis of the *AsGSs*. The 11 *AsGSs* were mapped to 5 chromosomes. The grey lines indicate all synteny blocks in the oat genome, and the duplicated gene pairs of *AsGSs* are connected with red lines.

To improve the knowledge of the functions of and mechanisms underlying *AsGS* gene family members in oat, fragment replication events were investigated via the MCScan toolkit using TBtools, and 42,965 collinear genes and 2,446 groups of tandemly duplicated genes were identified at the oat genome level. As shown in **Figure 3**, two gene pairs (AsGS1-2-1D/5A and AsGS1-2-1D/4C) were distributed on different chromosomes in six collinear gene pairs, and these were considered fragment duplication-derived genes. These results indicated that *AsGS* genes probably originated via gene duplication and that segmental duplication events played a significant role in *AsGS* evolution.

To further analyze the selective constraints among the *AsGS* genes in oat, we calculated the Ka/Ks ratio for the *AsGS* gene pairs. In the present study, the Ka values for each gene pair ranged from 0.001227 to 0.022700, whereas the Ks values ranged from 0.016022 to 0.136245 (**Supplementary Table S4**). All *AsGS* gene pairs with Ka/Ks values <1 were subjected to negative selection. Taken together, they were subject to purifying selection during evolution.

### 3.4 Regulatory elements in the *AsGS* promoters

**Figure 4:**
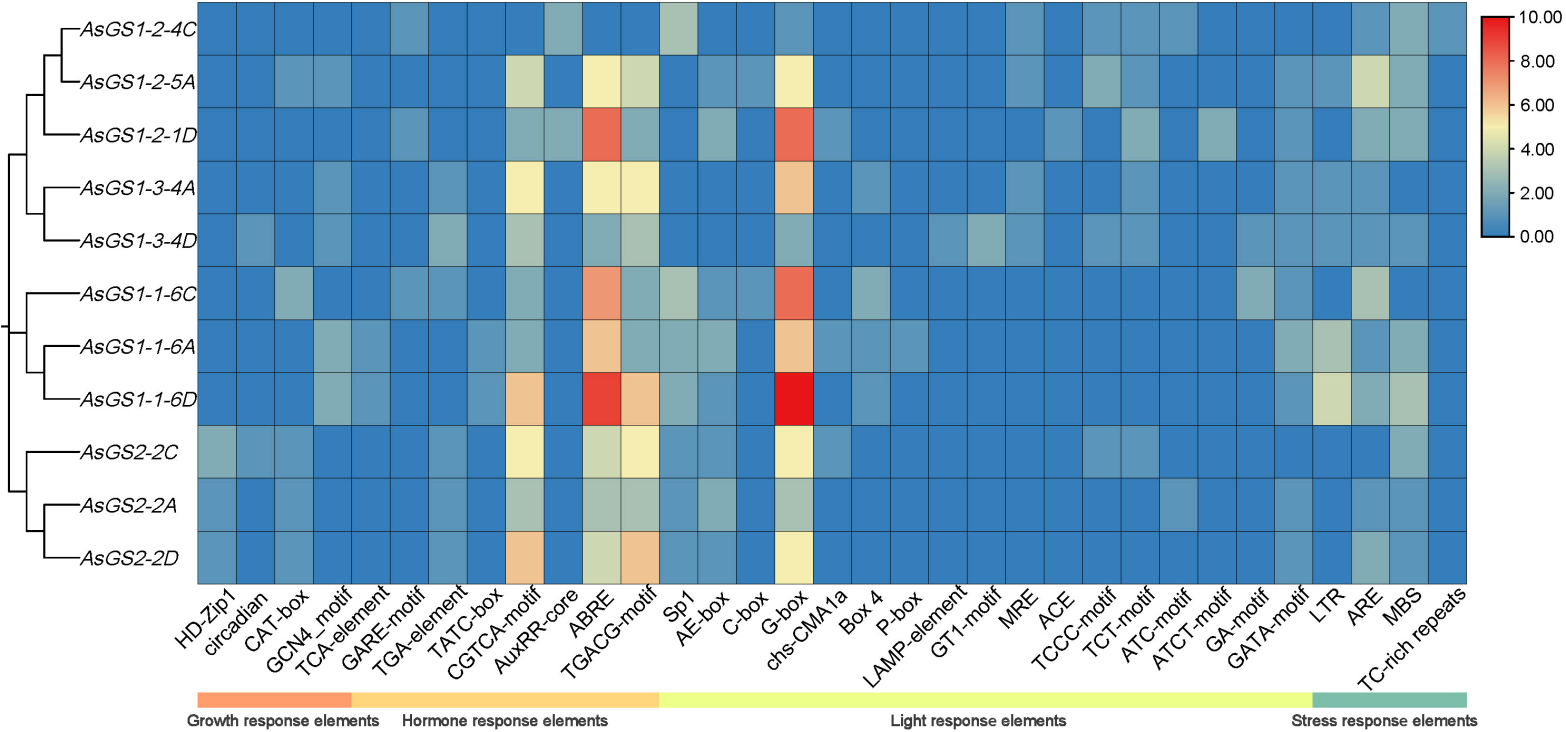
Cis-elements in putative promoter regions of AsGSs in oat. The colors of the boxes indicate the number of cis-acting elements.

To identify putative cis-elements involved in *AsGS* transcriptional regulation, the 2.0-kb promoter region upstream from the ATG translation start codon of each *AsGS* gene was analyzed. Thirty-three major putative cis-elements were selected and grouped into four categories, that is, elements responsive to stress, hormones, light and growth (**Figure 4**). The four stress-responsive cis-elements included LTRs (10), MBSs (16), AREs (18) and TC-rich repeats (1). Eight hormone-responsive elements, namely, TCA elements (2), GARE motifs (3), TGA elements (7), TATC boxes (2), CGTCA motifs (38), AuxRR-core (4), ABREs (53) and TGACG motifs (38), were detected in the promoter regions of AsGS genes. Moreover, 17 light-responsive elements, namely, Sp1 (13), AE boxes (11), C boxes (2), G boxes (59), chs-CMA1a (3), Box 4 elements (5), P box (1), LAMP element (1), GT1 motifs (2), MRE (4), ACE (1), TCCC motifs (5), TCT motifs (7), ATC motifs (2), ATCT motifs (2), GA motifs (3), and GATA motifs (10), were also found in the promoters of *AsGS* genes. In addition, the growth-responsive elements included HD-Zip 1(4), circadian (2), CAT boxes (6) and GCN4 motifs (7). Thus, we speculate that *AsGSs* may be involved in the abiotic stress response, light signaling and hormonal regulation and that different *AsGS* members of the same subfamily may have different response patterns.

### 3.5 Expression of *AsGSs* in response to abiotic stress in shoots and roots

**Figure 5:**
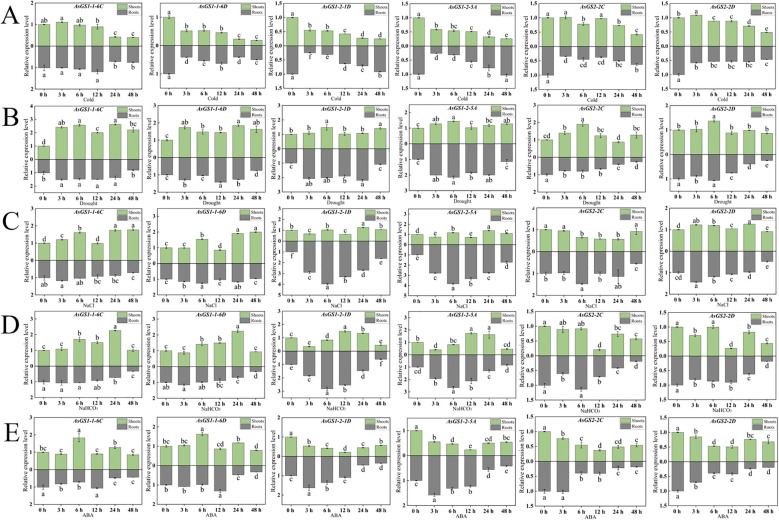
Analysis of expression patterns under abiotic stresses. **(A-E)** show the expression patterns of genes under cold, drought, salt, and alkali stresses and ABA treatment, respectively. The letters denote statistically significant differences (α=0.05).

To further clarify the potential functions of *AsGSs*, we performed qRT–PCR analysis of six *AsGSs* from different oat samples under various abiotic stresses and ABA treatment. The results revealed that the six selected *AsGSs* were expressed in the roots and shoots of oat plants and responded to abiotic stresses and ABA treatment with different expression patterns (**Figure 5**).

Most of the *AsGSs* were upregulated in shoots in response to drought, salt and alkali stresses (**Figures 5B–D**), whereas under cold stress and ABA treatment, most of the *AsGSs* presented downregulation of expression in shoots and roots (**Figures 5A, E**). Under cold stress, all the *AsGSs* exhibited the lowest expression at 48 h in the shoots, whereas all the *AsGSs* except *AsGS1-1-6C* presented a sharp decrease in expression at 3 h in the roots. Under drought stress, all the *AsGSs* except *AsGS1-1-6C* and *AsGS1-1-6D* presented the highest expression at 6 h in the shoots, whereas the expression of *AsGS2-2C* and *AsGS2-2D* tended to decrease with increasing stress duration in the roots. A comparison of the expression patterns among different subfamily members under the same stress treatment revealed that most members of the same subfamily presented similar expression patterns. The expression of *AsGS1-2-1D* and *AsGS1-2-5A* tended to increase first but then decreased under drought, salt, and alkali stresses and ABA treatment but not under cold treatment. *AsGS1-1-6C* and *AsGS1-1-6D* expression in the shoots peaked at 48 h under salt stress, at 24 h under alkali stress, and at 6 h under ABA treatment. Taken together, the results reveal that *AsGSs* can respond to abiotic stresses and ABA treatment and that the expression of the responsive genes vary over time. The expression pattern of *AsGS2-2C* was the most representative of the change in expression under abiotic stresses and ABA treatment: the relative expression of this gene was significantly upregulated under drought stress in shoots but was significantly downregulated in response to cold and drought stresses in shoots as well as in response to alkali stress and ABA treatment.

### 3.6 Isolation and characteristics of *AsGS2-2C*

**Figure 6:**
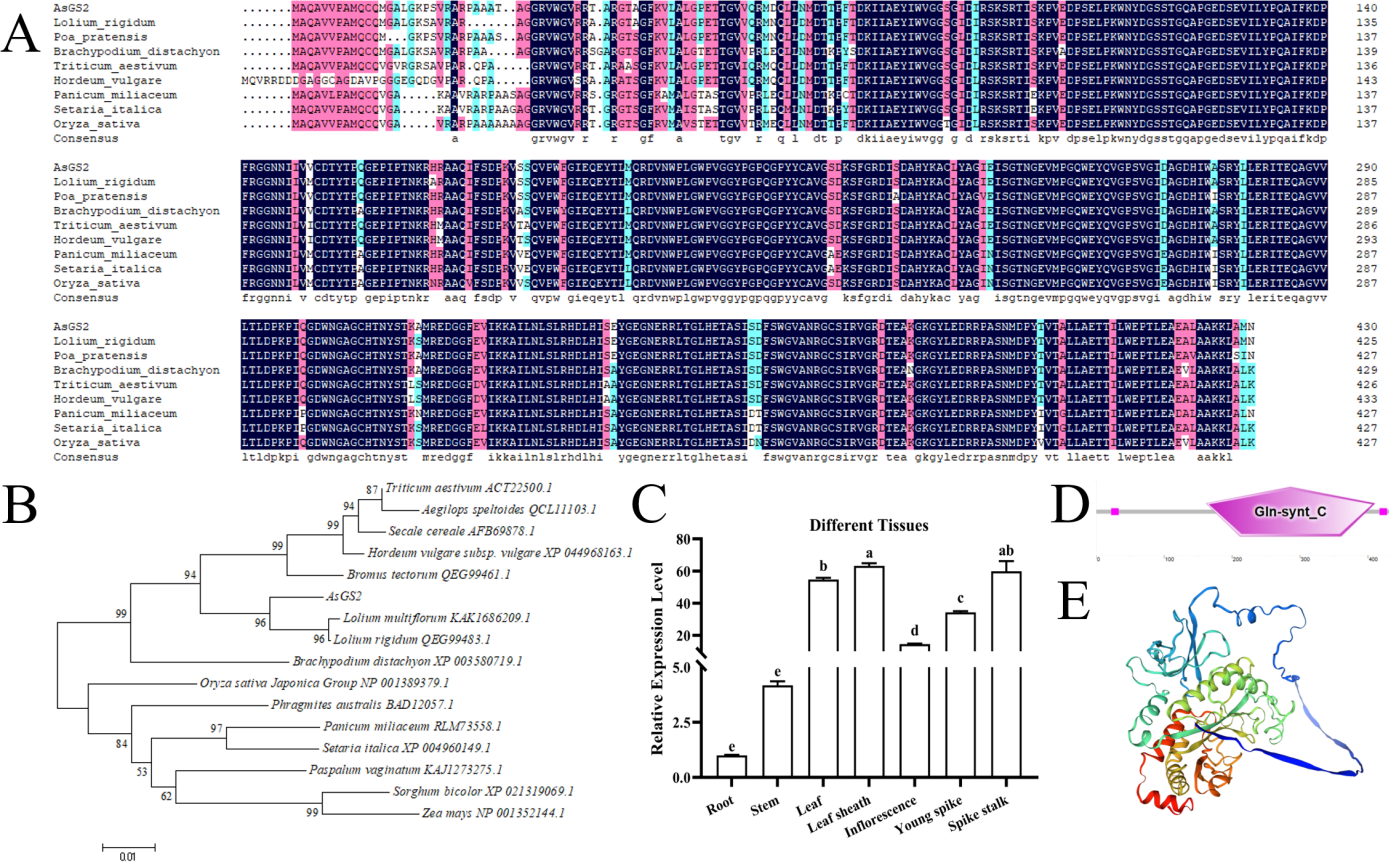
Amino acid sequence analysis of AsGS2-2C and expression pattern of coding genes. **(A)** Amino acid sequence alignment. **(B)** Phylogenetic tree of AsGS2-2C. **(C)** Expression level of the *AsGS2-2C* gene in oat at different tissue sites. **(D)** Domains of the AsGS2-2C protein. **(E)** 3D protein model of AsGS2-2C. The letters denote statistically significant differences (α=0.05).

On the basis of the above results regarding the expression patterns analysis, the collinearity analysis and the relative specificity for GS2 subfamily in the domain analysis, *AsGS2-2C* was cloned from oat leaves to further explore its function. The CDS of *AsGS2-2C* is 1,296 bp in length, encoding a 431-amino acid protein containing all 20 amino acids, including 44 positively charged amino acid residues (Arg, Lys), and 50 negatively charged amino acid residues (Asp, Glu) (**Figure 6A**). AsGS2-2C has a chemical formula of C_2079_H_3237_N_581_O_632_S_16_, a molecular weight (MW) of 47.00 kDa, and a theoretical isoelectric point (pI) of 5.76 and is a weakly acidic protein. The AsGS2-2C protein aqueous solution has an extinction coefficient of 84,715 at 280 nm, a grand average of hydropathicity of -0.375, and an instability index of 35.83, suggesting the stability of the protein. The AsGS2-2C protein, as accurately predicted by its three-dimensional structure, exhibits the following structural composition: 23.90% α-helices, 19.95% β-extension chains, and 56.15% irregular curls. This protein contains the Gln-synt_C domain (**Figures 6D, E**). According to the phylogenetic tree, AsGS2-2C was on the same branch as the GSs of *Lolium rigidum* and *Lolium multiflorum*, indicating that these genes shared the greatest similarity (**Figure 6B**). *AsGS2-2C* was highly expressed in the leaf sheaths, leaves, spike stalks and young spikes of oat and was expressed at the lowest level in the roots (**Figure 6C**).

### 3.7 Subcellular localization of AsGS2-2C

**Figure 7:**
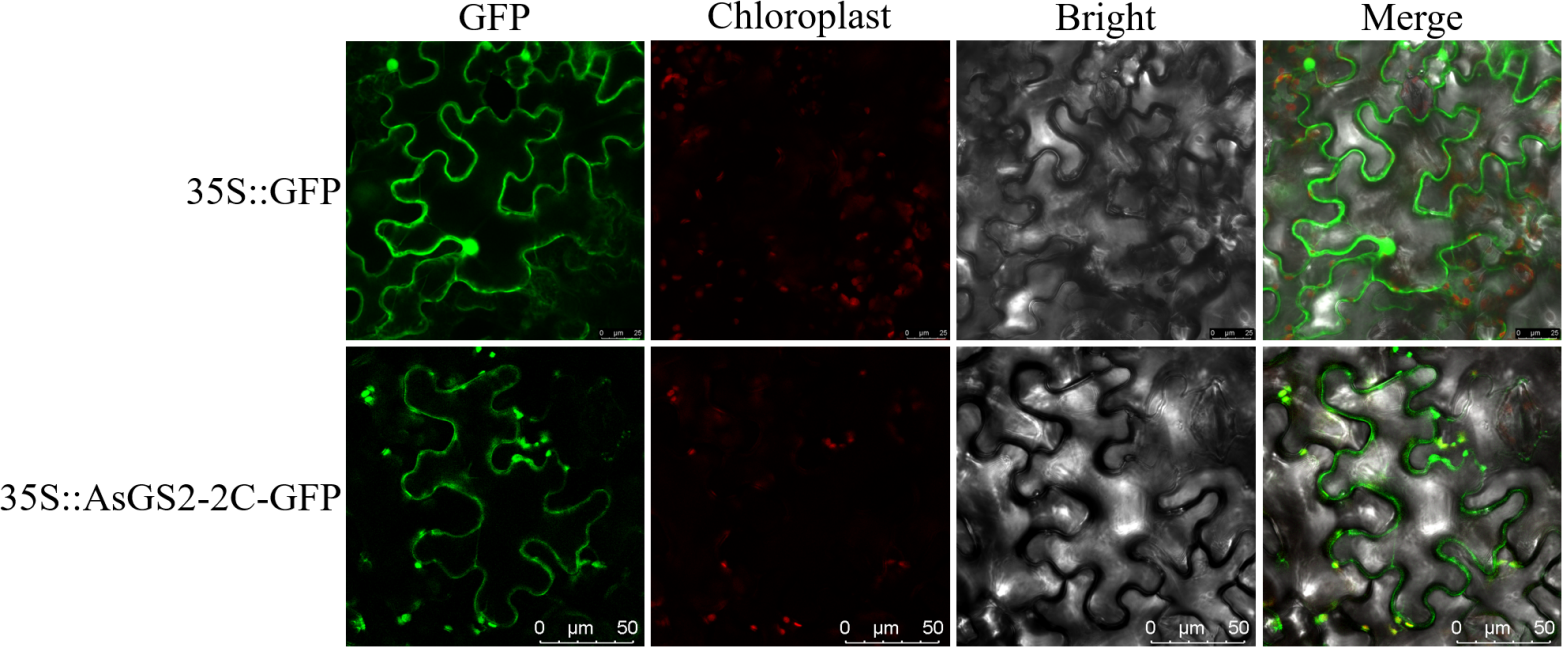
Subcellular localization of AsGS2-2C.

To investigate the subcellular distribution of AsGS2-2C, an AsGS2-2C-GFP fusion protein was transiently coexpressed in *N. benthamiana* leaves and then visualized using laser scanning confocal microscopy. The fluorescence signal of 35S::GFP is mainly in the cell nucleus and cell membrane, however AsGS2-2C-GFP fluorescence was detected in the cell membrane and chloroplasts, as evidenced by the bright field image of infected leaves, as well as the merged image (**Figure 7**). We think that AsGS2-2C protein is located on chloroplasts and cell membrane.

### 3.8 The ability of transgenic plants to tolerate drought is enhanced by *AsGS2-2C* upregulation

**Figure 8:**
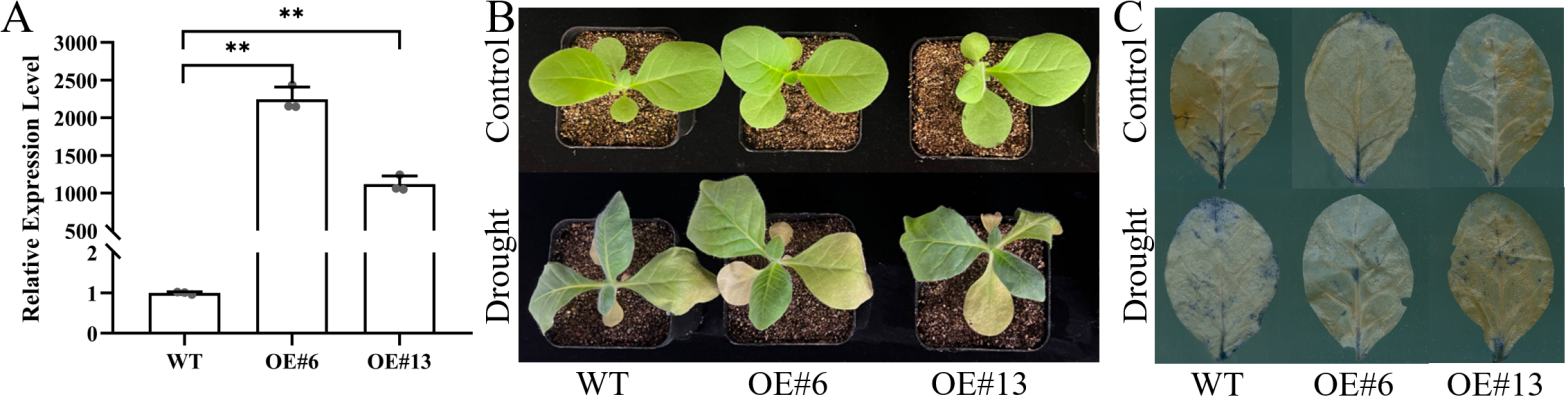
Identification and phenotype of AsGS2-2C-transformed lines. **(A)***AsGS2-2C* relative expression levels in the transgenic tobacco lines. **(B)** Phenotypes of transgenic tobacco plants overexpressing *AsGS2-2C* under control condition and drought stress. **(C)** Images of transgenic tobacco plants overexpressing *AsGS2-2C* stained with NBT staining solution under control conditions and drought stress. The means ± standard deviations from three biological replicates are shown with error bars. “**”: extremely significant difference between the WT and transformation lines (*P*<0.01). WT: wild type; OE#6 and OE#13: transgenic tobacco lines 6 and 13, respectively. The same applies below.

To investigate the response of *AsGS2-2C* to drought stress, transgenic tobacco (T2) plants were created and validated with *Bar* (**Supplementary Figure S3**). The *AsGS2-2C* transcript levels in the OE#6 and OE#13 transgenic lines were 2,246.1 and 1,120.2 times greater than those in the WT (**Figure 8A**). No phenotypic difference was detected between AsGS2-2C-transformed and WT plants under normal conditions. After being exposed to drought for six days, the leaves of the WT and *AsGS2-2C* transgenic lines showed wilting and chlorosis, and the symptoms were more severe in the WT leaves. After 10 days of drought stress, the leaves of all the lines were severely chlorotic, and the WT plants wilted more severely (**Figure 8B**). This finding was consistent with the images of O_2_^-^ stained with NBT staining solution (**Figure 8C**). The results demonstrated that the drought resistance of the transgenic plants was increased by overexpressing *AsGS2-2C*.

### 3.9 Modifications in the physiology of transgenic plants overexpressing *AsGS2-2C* under drought stress

**Figure 9:**
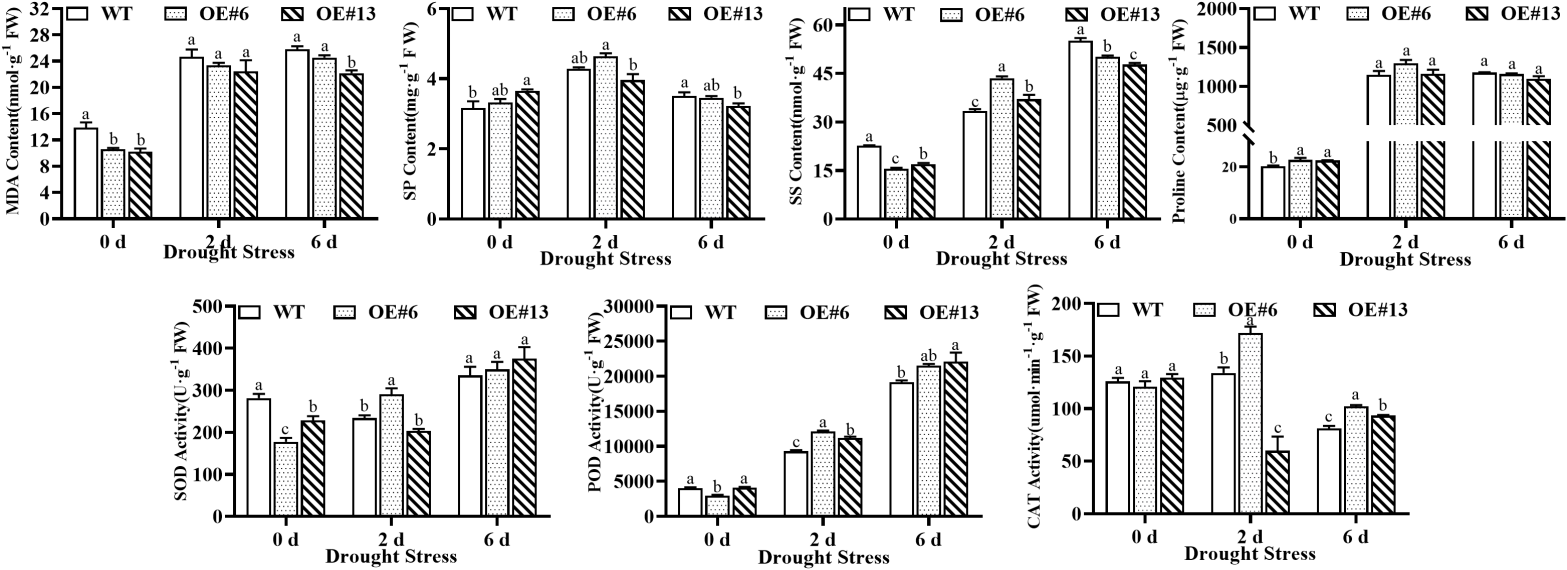
Modifications in the physiology of transgenic tobacco plants overexpressing *AsGS2-2C* under drought stress. The letters denote statistically significant differences (α=0.05). MDA, malondialdehyde. SOD, superoxide dismutase. POD, peroxidase. CAT, catalase. SP, soluble protein. SS, soluble sugar.

To better demonstrate the role of *AsGS2-2C* in stress tolerance, the physiological modifications in transgenic tobacco plants subjected to drought stress were studied. Compared with those of the WT, the proline contents of the transgenic tobacco lines increased in response to *AsGS2-2C* upregulation (**Figure 9**). With increasing stress duration, the POD activity of each line increased significantly, and that of OE#13 peaked at 6 days of stress. The CAT activity of all the transgenic lines was significantly greater than that of the WT after 6 days of drought stress, especially that of OE#6, which peaked (171.88 µmol·min^-1^·g^-1^ FW) at 2 days of stress. Even though the SOD activity of the transgenic tobacco was lower than that of the WT tobacco in the control environment, OE#6 showed significantly increased SOD activity after being subjected to drought treatment. The contents of MDA in the transgenic lines were significantly lower than those in the WT plants under normal conditions and after 6 days of drought stress. However, the contents of SS and SP in the transgenic lines were significantly greater than those in the WT plants under stress for 2 d, but these contents were significantly lower than those in the WT plants under stress for 6 d.

### 3.10 Expression analysis of genes associated with the overexpression of *AsGS2-2C* in transgenic plants under drought stress

The expression patterns of 12 genes, namely, *MnSOD*, *Cu/Zn-SOD*, *CBL1*, *GAPC*, *GR1*, *Gln1-5*, *BI-1*, *ACR11*, *GLU*, *ERD10B*, *Hxk3*, and *Ltp1*, were assessed in the transgenic and WT plants via qRT–PCR. The overexpression of *AsGS2-2C* significantly increased the expression levels of 12 associated genes under normal conditions. The expression of *Cu/Zn-SOD*, *MnSOD*, *CBL1*, *GR1*, *GAPC*, *Gln1-5*, and *BI-1* significantly increased in the transgenic lines compared with the WT plants under drought stress for 2 d and 6 d. However, the expression of *ACR11*, *GLU*, *ERD10B*, *Hxk3* and *Ltp1* in the transgenic lines significantly increased at two days of drought stress and significantly decreased at 6 days of drought stress.

## 4 Discussion

Drought significantly constrains crop yield, poses a threat to global food security, and adversely impacts plant growth and development (Vadez et al., 2024). Investigating drought-related genetic resources across various plant species through molecular techniques has improved our understanding of plant drought responses (Yang and Qin, 2023). GSs serve as pivotal enzymes in the initial step of NH_4_^+^ assimilation, facilitating the synthesis of glutamine. Despite some research on the role of GS genes in nitrogen uptake and utilization (Martin et al., 2006a; Li et al., 2011; Hachiya et al., 2021), there is a lack of research on the identification of *GSs* in oat and their role in the stress response. Consequently, in this study, the *AsGS* gene family in oat was identified, and bioinformatic and expression pattern analyses of these genes were performed under diverse stress conditions. These findings contribute to a deeper understanding of oat *AsGSs* and offer valuable insights into their functional roles.

In this study, 11 *AsGS* genes were identified in oat, of which 4, 3, and 4 *AsGS* genes were in the A, C, and D subfamilies, respectively (**Supplementary Table S2**), similar to the numbers of *GS* genes in *Arabidopsis* (6) (Li et al., 2006), rice (4) (Mondal et al., 2021), and wheat (12) (Wei et al., 2022). Phylogenetic trees were constructed using the sequences from oat, wheat, *Arabidopsis* and rice (**Figure 1**), and the oat *AsGS* genes were clustered into four subfamilies: GS1-1, GS1-2, GS1–3 and GS2. Oat, rice, and wheat are monocots, and members of each subfamily are closely related to each other and clustered on the same branch. However, *Arabidopsis* is a dicot plant and did not cluster closely with oat, wheat, or rice, indicating that *GS* genes differ among species and that the divergence of *GSs* may have occurred before the divergence between monocots and dicots (Bernard and Habash, 2009). Previous studies have shown that the activity of the rice GS1 enzyme is stronger than that of the *Arabidopsis* (Ishiyama et al., 2004), and this result supports the idea that *GSs* differ between monocots and dicots in our clustering results. *OsGS2* in rice is relative conservative during evolution (Jiang and JingLiu, 2007). Collinearity analysis also confirmed the conservation of *AsGSs* in oat (**Figure 3**). Members within the same subtype presented similar sequence lengths, physicochemical properties, gene structures, and motif distributions. In contrast, variations in these features were noted across different subtypes, with the most pronounced differences observed between GS2 and the other subtypes (**Supplementary Table S2**; **Figure 2**). This observation may be attributed to the distinct characteristics of the GS2 and GS1 domains. Although GSs are highly conserved across plant species, interspecies variations exist, which warrants further investigation into whether these differences contribute to novel GS functions in oat. Additionally, repetitive events in active regions, CDSs, regulatory sequences, and promoter regions may facilitate the acquisition of new functions by gene family members (Abdullah et al., 2021; Musavizadeh et al., 2021).

The promoter region of *AsGSs* is abundant with numerous response elements (**Figure 4**). Analyzing the expression patterns of *AsGSs* offers valuable insights into the potential functions of the *AsGS* gene family. The results indicated that six oat *AsGS* genes were significantly downregulated under cold stress, but *AsGSs* presented a positive response to other abiotic stresses and ABA treatment. Notably, the expression patterns of *AsGSs* varied significantly across the different treatments (**Figures 5A–E**). Specifically, *AsGSs* presented the most pronounced response to drought and salt stress. Under drought conditions, *AsGS* expression was markedly upregulated in both aboveground and root tissues (**Figure 5B**). In response to salt stress, the upregulation of the expression level of *AsGSs* in roots was significantly greater than that in aboveground tissues (**Figure 5C**). These findings are consistent with the results of Debouba et al (Debouba et al., 2006). In the maize inbred line *B73*, *gln2* and *gln6* were identified as genes responsive to drought stress (He et al., 2020). The *ZmGln1–3* gene was upregulated under drought conditions, which altered the plant nitrogen distribution (Li et al., 2016). The expression of *OsGS1;1* in leaves was elevated under moisture deficiency, with lower transcription levels observed in the stems and roots of rice. The reduction in GS activity in the leaves of drought-sensitive cultivars was attributed primarily to decreased GS2 activity (Singh and Ghosh, 2013). Additionally, NaCl treatment was found to reduce the expression of *GLN2* and *GLN1;2* in the leaves of Arabidopsis thaliana but significantly upregulated *GLN1;1* and *GLN1;2* transcription in roots (Debouba et al., 2013). These suggest that GSs in various species respond differently to stress, with expression levels varying across different tissues. Our findings revealed that members of the oat *AsGSs* exhibit distinct mechanisms of action when subjected to identical stress conditions.

To explore the function of *AsGSs*, we comprehensively consider the results of bioinformatics analysis and the expression patterns analysis, and selected *AsGS2-2C*, first performing subcellular localization analysis. The AsGS2-2C GFP signal was localized to the chloroplast and cell membrane (**Figure 7**). This finding is different from previous finding of the chloroplast localization the GS2 gene; GS1 is localized in the cytoplasm, whereas GS2 is localized mostly in the plastid (Tjaden et al., 1995). The presence of a membrane signal suggests a potential secondary localization or an association with the chloroplast envelope membranes, which warrants further investigation. Next, the *AsGS2-2C* gene was introduced into tobacco plants to assess its functional impact on drought tolerance. Following exposure to drought stress, the degree of wilting and the development of NBT coloration in AsGS2-2C-overexpressing plants demonstrated that this gene positively regulates plant drought tolerance (**Figure 8D**). In drought-tolerant wheat varieties, the activity of GS2 isoenzymes remains unchanged, whereas in drought-sensitive varieties, there is a significant reduction in GS2 isoenzyme activity, accompanied by premature leaf senescence (Nagy et al., 2013). The observed phenotypic differences in plants subjected to drought stress in this study align with findings from comparative analyses of drought-tolerant and drought-sensitive wheat cultivars. ROS are byproducts of normal plant cell metabolism, and their balance is disrupted under drought stress. ROS can induce the degradation of pigments, proteins, lipids, and nucleic acids, leading to the rapid inactivation of enzymes, the destruction of organelles and cell membranes, and ultimately resulting in cell death (Sahu et al., 2022).

Plants possess intricate enzymatic and nonenzymatic antioxidant defense mechanisms that safeguard cells from oxidative damage and facilitate the removal of metabolically generated ROS (Karuppanapandian et al., 2011). In this study, the activities of POD and CAT were markedly greater in *AsGS2-2C* transgenic lines than in WT plants after six days of drought stress. These findings suggest that the overexpression of *AsGS2-2C* enhances resistance to ROS-induced damage under drought conditions by increasing the activities of antioxidant enzymes. The plasma membrane (PM) is a biological membrane that separates the inside of all cells from the outside (Cassim et al., 2019). Under stress conditions, damage to the plant cell membrane system can increase the permeability of the plasma membrane, leading to the leakage of soluble substances and small organic molecules from the cells, thereby causing metabolic disturbances (Wang et al., 2020). MDA serves as a crucial indicator of membrane damage under abiotic stress, reflecting the extent of cellular injury (Ayala et al., 2014). In our study, the MDA content increased across all the lines subjected to drought stress for six days. However, the MDA content in the *AsGS2-2C* transgenic lines remained significantly lower than that in the WT, suggesting that overexpression of *AsGS2-2C* mitigated drought stress by reducing cell membrane damage. Osmotic regulation is crucial for maintaining crop yield and plays a significant role in plant adaptation to drought and dehydration (Blum, 2017). Overexpression of the wheat *GS1* and *GS2* genes in tobacco has been shown to increase drought tolerance by increasing sucrose, proline, and chlorophyll accumulation, as well as the ROS scavenging capacity (Yu et al., 2020). In this study, tobacco plants overexpressing *AsGS2-2C* presented a marked increase in soluble sugar and protein contents after two days of drought stress. However, a significant decrease was observed after six days of drought stress, which may be attributable to variations in stress severity. In conclusion, AsGS2-2C-overexpressing plants respond to drought stress through the regulation of osmoregulatory substances. However, the physiological mechanism underlying the drought response was predominantly associated with alterations in cell membrane permeability and the activity of the antioxidant enzyme system.

*Cu/ZnSOD* and *MnSOD* are regarded as the first line of defense within the antioxidant enzyme system in most organisms (Zelko et al., 2002). The *GR1* gene, which encodes an antioxidant enzyme, plays a critical role in the ascorbic acid–glutathione (AsA–GSH) cycle, effectively maintaining GSH levels to mitigate oxidative stress (Gill et al., 2013). *GAPC* is involved in the plant response to oxidative stress, arising from both abiotic and biotic factors, and is recognized as a pivotal enzyme in the glycolysis and gluconeogenesis metabolic pathways (Danshina et al., 2001; Zhang et al., 2011, 2020). Our findings demonstrated that the expression levels of *Cu/ZnSOD*, *MnSOD*, *GR1*, and *GAPC* were significantly elevated in tobacco plants overexpressing *AsGS2-2C* following drought stress. Furthermore, *AsGS2-2C* confers protection to oat plants under severe drought by modulating the oxidative stress response and engaging the antioxidant enzyme system.

Fd-GOGAT is a crucial enzyme involved in the initial stages of ammonia assimilation in plants (Dincturk and Knaff, 2000). The *GLU* gene encodes the Fd-GOGAT protein, and its expression in mesophyll and vascular cells indicates that Fd-GOGAT plays a role in photorespiration and provides amino acids for nitrogen transport (Feraud et al., 2005). Our findings demonstrate that the overexpression of *AsGS2-2C* enhances the expression level of *GLU* in transgenic plants under both normal conditions and drought stress for two days. *AsGS2-2C* overexpression can modulate the Fd-GOGAT protein to improve nitrogen transport, thereby sustaining physiological activities under normal conditions and during the early stages of drought stress. However, after six days of drought stress, the *GLU* levels in the transgenic lines were significantly lower than those in the WT, suggesting the presence of a more complex regulatory mechanism under prolonged drought stress. Additionally, ACT-domain-containing family protein (ACR11) localizes to chloroplasts and interacts with Fd-GOGAT (Takabayashi et al., 2016). *ACR11* functions as an activator of *GS2*, thereby playing a crucial role in nitrogen assimilation in *Arabidopsis* (Osanai et al., 2017). In this study, the expression level of *ACR11* was comparable to that of the GLU gene, indicating that *AsGS2-2C* may simultaneously mediate the potential interaction between *ACR11* and *GLU* in response to drought stress. Both the GS1 and GS2 isozymes are forms of GS, yet they differ in their nitrogen reutilization sites. GS1 primarily operates in the leaf vein, whereas GS2 is active in mesophyll cells (Moison et al., 2018). Nevertheless, some research has demonstrated that although the Arabidopsis *GS2* mutant exhibits reduced size, weak chlorosis, and impaired nitrogen metabolism, it is still capable of growing and completing its life cycle under normal conditions. This finding suggests that the absence of *GS2* can be compensated for by the overexpression of the cytoplasmic GS genes *GLN1;2* and *GLN1;3* (Ferreira et al., 2019). In this study, the expression of *Gln1–5* in AsGS2-2C-overexpressing tobacco plants was significantly elevated compared with that in WT plants under normal conditions. With increasing drought stress duration, the expression level of *Gln1–5* in all the lines tended to decrease, but the expression level of *Gln1–5* in the *AsGS2-2C* transgenic lines was still significantly greater than that in the WT, indicating that the overexpression of the *AsGS2-2C* gene could promote the expression of *Gln1–5* in tobacco. The indicated coordinated cross-compartmental enhancement among them, and the synergistic regulation of nitrogen metabolism still needs further study.

**Figure 10:**
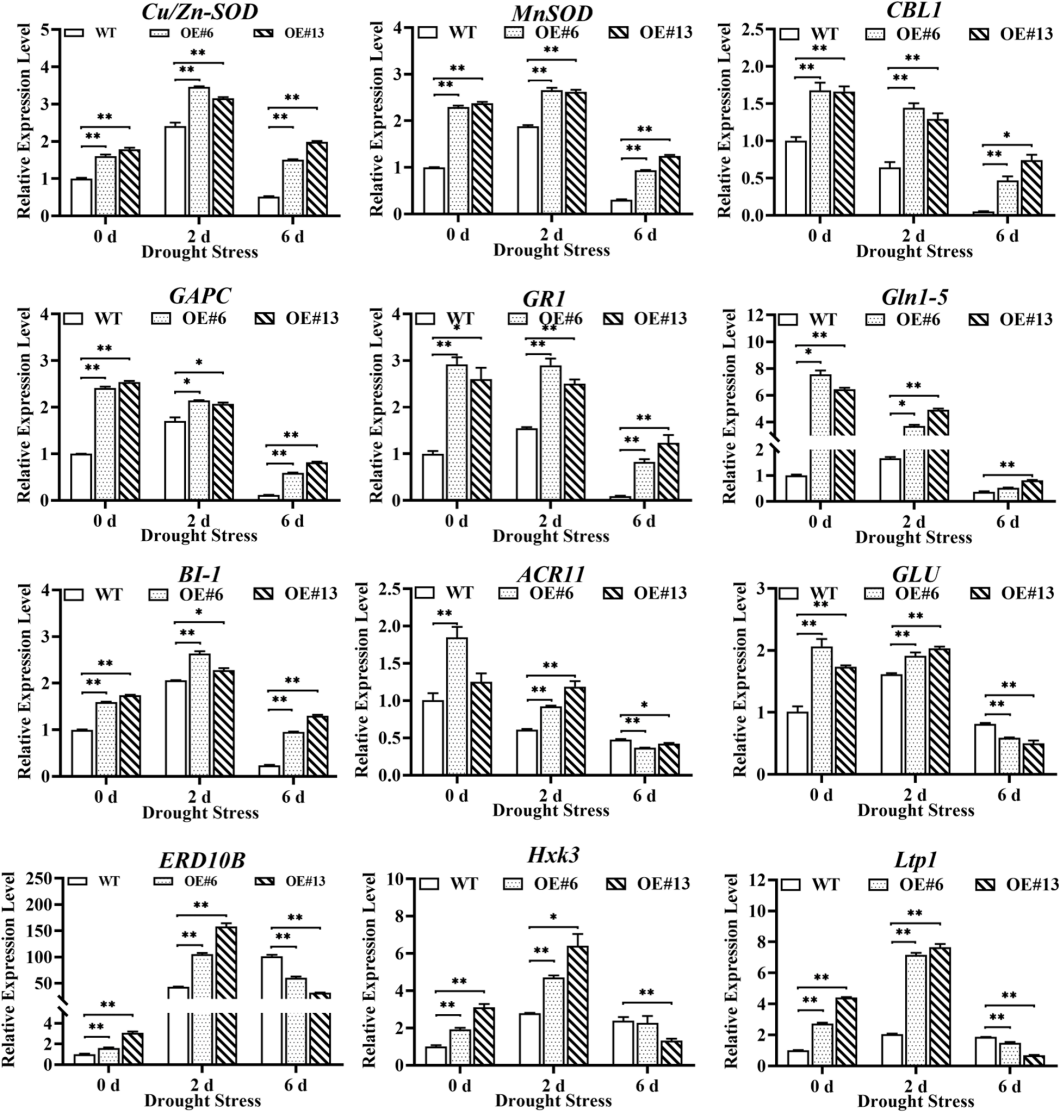
Expression of 12 associated genes in transgenic plants overexpressing *AsGS2-2C* under drought stress. “**” and “*”: extremely significant difference (*P*<0.01) and significant difference (*P*<0.05) between the WT and transformation lines. The letters denote statistically significant differences (α=0.05).

**Figure 11:**
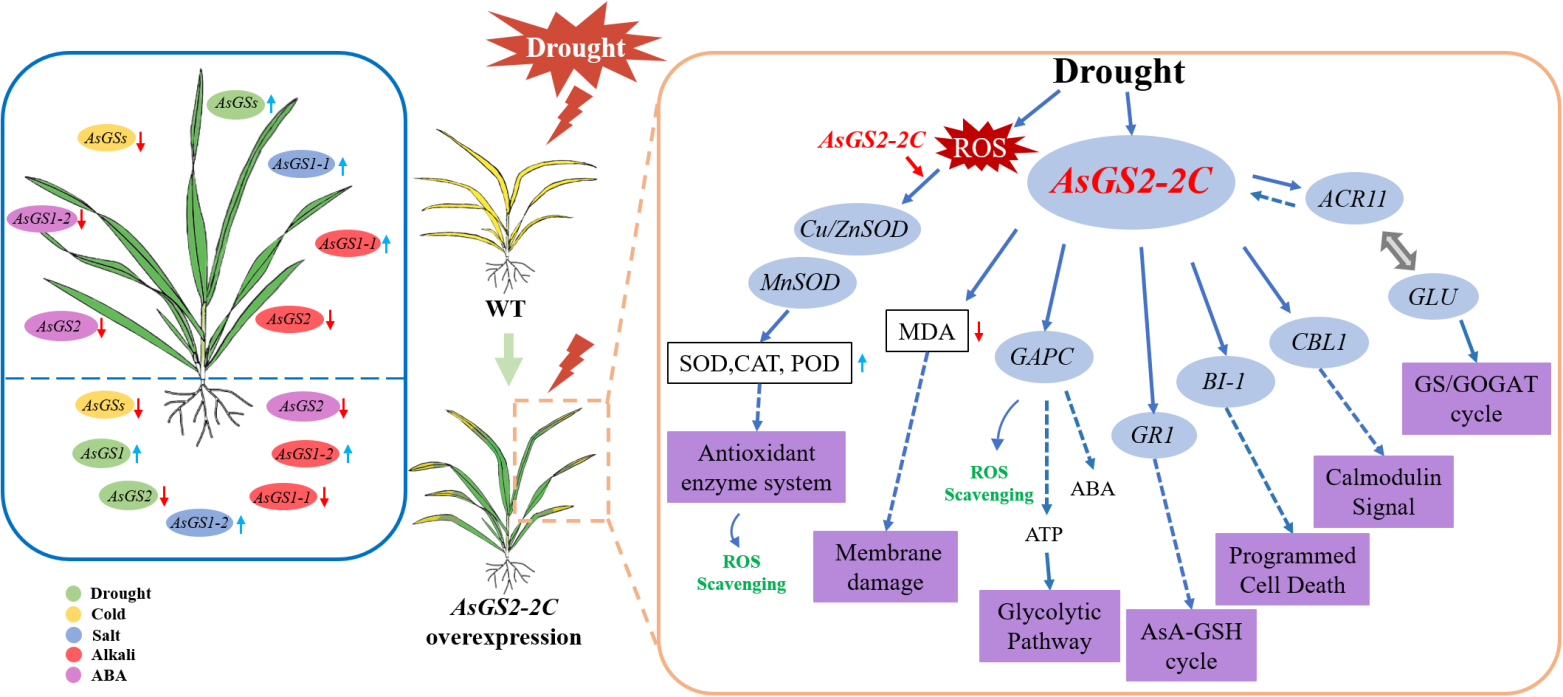
Schematic model of the role of *AsGSs* stress tolerance and *AsGS2-2C* response to drought.

Calcineurin B-like 1 (CBL1), a calcium sensor, is known to positively regulate responses to drought stress (Cheong et al., 2003). Bax inhibitor-1 (BI-1) is a conserved protein associated with the endoplasmic reticulum (ER) that serves a cytoprotective function and interacts with various proteins to modulate ER stress, oxidative stress, and cellular Ca^2+^ depletion (Kawai-Yamada et al., 2009). In this study, despite the overall downregulation of *BI-1* gene expression, the expression levels of the *CBL1* and *BI-1* genes in the transgenic lines were significantly greater than those in the WT lines under continuous drought stress (**Figure 10**). These findings suggest that the *AsGS2-2C* gene may increase the expression of *CBL1* and *BI-1* under drought conditions, thereby exerting a positive regulatory effect. Hexokinases (HXKs) were the first identified intracellular glucose sensors in plants, facilitating glucose phosphorylation and metabolism (Li and Sheen, 2016). Lipid-transfer proteins (LTPs) are secreted proteins that play crucial roles in fatty acid binding and the transfer of phospholipids and lipids between membranes *in vitro* and are implicated in various physiological processes related to plant growth, development, and responses to stresses (Rojas et al., 2019). Dehydrins exhibit properties akin to those of molecular chaperones, functioning as structural stabilizers that safeguard nuclear and cytoplasmic macromolecules against condensation during dehydration (Close, 2010). The expression of dehydrin *ERD10B* is modulated in response to ionic and osmotic stress and plays a role in oxidative stress processes (Gupta et al., 2014; Khedia et al., 2018). Our experimental findings revealed that the expression levels of *Hxk3*, *ERD10B*, and *Ltp1* in overexpressing plants were significantly elevated compared with those in WT plants after two days of drought stress. However, these parameters were markedly reduced in the transgenic plants after six days of stress. These suggest that the overexpression of *AsGS2-2C* can modulate plant growth and development under drought stress by influencing the tricarboxylic acid (TCA) cycle, lipid metabolism, osmotic regulation, and other physiological processes (**Figure 11**). The regulation of plant responses to stress is a highly complex process. Plants exhibit heightened stress resistance after two days of stress, but their functional capacity diminishes with prolonged stress exposure. Consequently, the physiological states of the transgenic plants differed between two and six days of stress, warranting further investigation.

The studies on the functional verification of genes have been conducted in various species such as rice (Kang and An, 2005), alfalfa (Sun et al., 2019; Yin et al., 2022b), *Gossypium hirsutum* (Yang et al., 2024), and tea plants (He et al., 2023) by using genetic transformation of tobacco. This further demonstrates the credibility of the functional verification results of *AsGS2-2C* in transformed tobacco. However, there are still certain limitations, as the biological characteristics and genetic backgrounds of tobacco and oat are not completely same. In addition, the use of expression vectors with GFP tags for overexpression may have a potential impact on the stability of proteins in positive lines. We will continue to construct an overexpression vector without other tags, and explore the function of *AsGS2-2C* in transformed original plants and conduct comparative analysis. Currently, it has been confirmed that *AsGS2-2C* can enhance the drought resistance of plants, providing genetic resources for molecular breeding design. The *GS* gene plays a certain role in nitrogen assimilation efficiency. We will continue to develop molecular markers for high nitrogen utilization rate, high yield, and drought tolerance to further explore the related mechanisms.

## 5 Conclusion

In summary, 11 *AsGS* genes were identified in the oat genome and were further classified into four subtypes and 2 subfamilies. Members of the same subtype presented highly conserved domain structures and motif compositions. The cis-acting elements of *AsGSs* responder involved in the response to abiotic stress, hormones and light. *AsGS* genes play pivotal roles in oat responses to abiotic stress and ABA signal transduction processes, particularly drought stress. The positive effect of *AsGS2-2C* on the enhancement of drought stress tolerance was determined on the basis of phenotype analysis and NBT staining. Overexpression of *AsGS2-2C* reduced plant wilting and led to increased antioxidant enzyme activity and reduced membrane damage by increasing POD and CAT activities and decreasing the MDA content. Simultaneously, *AsGS2-2C* mediated signal transduction by upregulating the transcription of *Cu/Zn-SOD*, *MnSOD*, *CBL1*, *GR1*, *GAPC*, *Gln1-5*, and *BI-1* and first upregulated and then downregulated the transcription of *ACR11*, *GLU*, *ERD10B*, *Hxk3* and *Ltp1* in transgenic plants under drought stress. Our findings elucidate the mechanism by which *AsGS2-2C* alleviates plant drought stress and suggest that *AsGS2-2C* should be incorporated into successful breeding initiatives to enhance the tolerance of oat to drought.

## Data Availability

The gene sequence is available in the NCBI, with GenBank accession No. PQ720318. All data generated or analyzed during this study are included in this published article and its **Supplementary Files**.
