# Supplementary material for: Identification and characterization of the glutamine synthetase gene family in oat (Avena sativa L.) and the role of AsGS2-2C under drought stress
Source: Front Plant Sci. 2025 Dec 1;16:1719654. doi: 10.3389/fpls.2025.1719654 (PMC12703710; doi:10.3389/fpls.2025.1719654)
Supplement: Supplementary file 1 [file DataSheet1.docx]

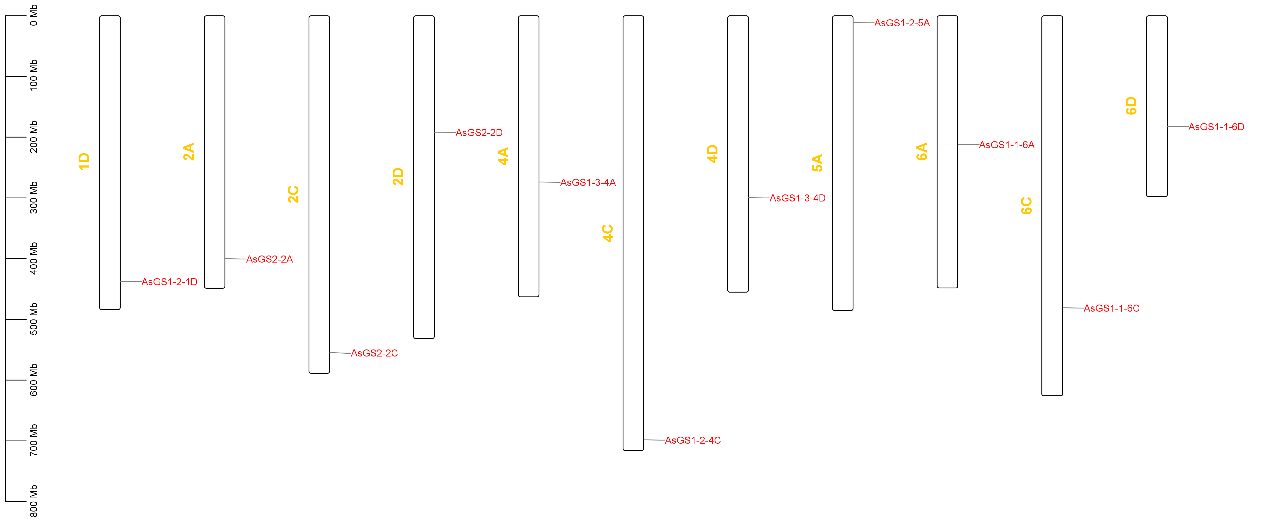


**Figure S1** The distribution of 11 *AsGS* genes on oat chromosomes.


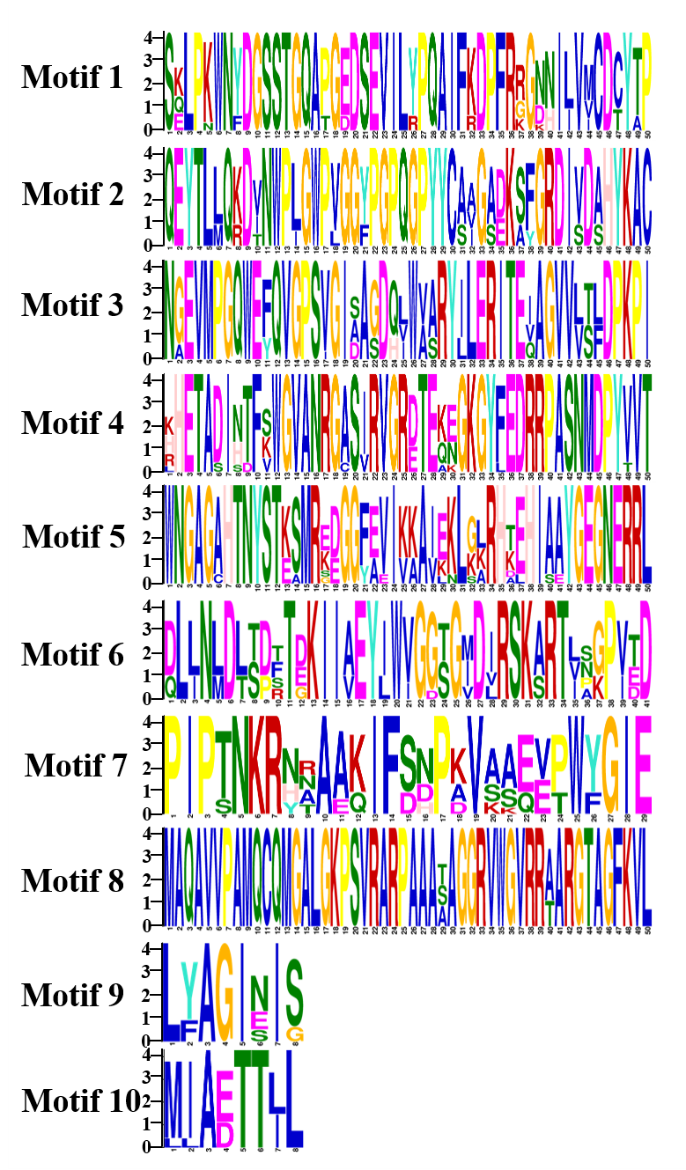


**Figure S2** Logos of 10 motifs in oat.


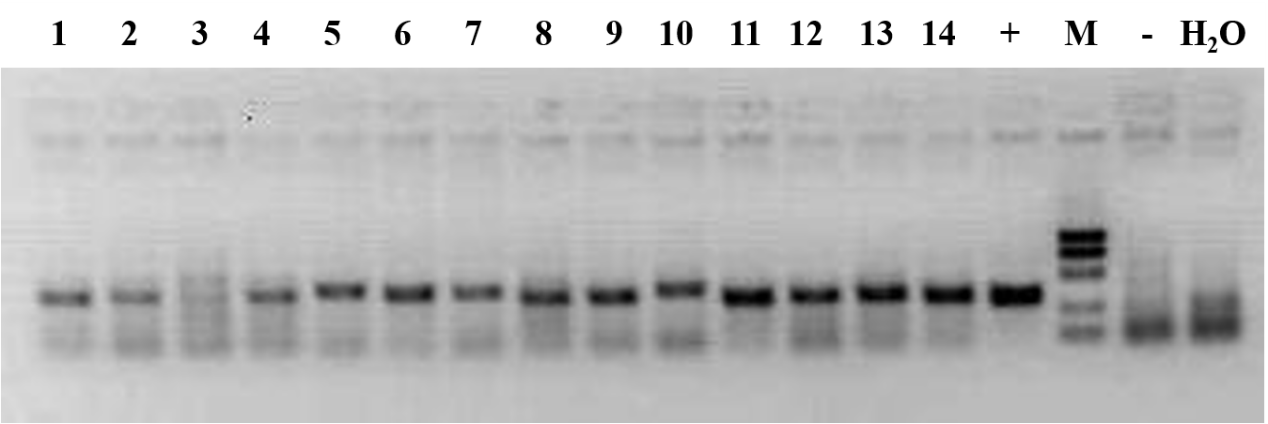


**Figure S3** Electrophoretic gel chart for identifying transgenic tobacco lines with *Bar* via PCR.
