# Supplementary material for: Identification and characterization of the glutamine synthetase gene family in oat (Avena sativa L.) and the role of AsGS2-2C under drought stress
Source: Front Plant Sci. 2025 Dec 1;16:1719654. doi: 10.3389/fpls.2025.1719654 (PMC12703710; doi:10.3389/fpls.2025.1719654)
Supplement: Supplementary file 2 [file Table1.docx]

**Table S1** List of primers for PCR in this study.

| **Primer name** | **Sequence (5' to 3')** |
| --- | --- |
| qPCR-AsGS1-1-6C-F | CTGAGATTGCCGGAGTTGTT |
| qPCR-AsGS1-1-6C-R | GACTCGGTGCTGTAGTTTGT |
| qPCR-AsGS1-1-6D-F | CCTGCAGAAGGATATCAACTGG |
| qPCR-AsGS1-1-6D-R | ACGACTTCTCAGCACCAATAC |
| qPCR-AsGS1-2-1D-F | GGGTGACCACATCCTTGTTAT |
| qPCR-AsGS1-2-1D-R | TGGCAGCATTGTTCCTCTT |
| qPCR-AsGS1-2-5A-F | GGGTGACCACATCCTTGTTAT |
| qPCR-AsGS1-2-5A-R | AGATCTTGGCGGCATTGT |
| qPCR-AsGS2-2C-F | GAGCTACCCAAGTGGAACTATG |
| qPCR-AsGS2-2C-R | CGGAACGGGTCCTTGAATATAG |
| qPCR-AsGS2-2D-F | CGTGGGATCAGACAAGTCATT |
| qPCR-AsGS2-2D-R | CTGATCTCGATTCCAGCGTAAA |
| qPCR-UBC2-F | ACAGCGTTCCGAGAGTTGTT |
| qPCR-UBC2-R | AAGAGTCAGCCCGAATGCAG |
| AsGS2-2C-F | ATGGCGCAGGCGGTG |
| AsGS2-2C-R | TCATACGTTCATCGCCAGCT |
| Bar-F | TCATGCCAGTTCCCGTGCTT |
| Bar-R | GGTCTGCACCATCGTCAACCA |
| 0-F | gagagaacacgggggactttgcaacatggcgcaggcggtggtgc |
| 0-R | cggccgctgtacatacgttcatcgccagcttcttggcagc |
| 1-F | ggcgatgaacgtatgtacagcggccgcttcagggag |
| 1-R | agagttcctcgcccttcacgatacaaattgctgcggcagccgatccagagc |
| NtActin-F | TTTGAGACTTTCAATGTGCCCGCC |
| NtActin-R | TAGCATGTGGGAGTGCATAACCCT |
| qPCR-CBL1-F | GCCTCACAAGAAGAGAAGATCA |
| qPCR-CBL1-R | AACTTCATCTCAGACTCGCATAG |
| qPCR-GAPC-F | GTGCTGATTTCGTTGTGGAATC |
| qPCR-GAPC-R | GAGCAGAGATCACAACCTTCTT |
| qPCR-BI-1-F | CTTTGGAACATTGGTGGCTTAC |
| qPCR-BI-1-R | CCATCAGAAGTGCTATCCTCTTT |
| qPCR-Hxk3-F | TTCATCAACCTCCTGGTAAGC |
| qPCR-Hxk3-R | CCTTTCGTCCACCTGATAAGAG |
| qPCR-GR1-F | CAGAATGACCAGAGTCCTCTAAC |
| qPCR-GR1-R | CCAGCACTTCATGTTTGTCTTC |
| qPCR-Cu/Zn-SOD-F | AGCAGCAGTGAAGGTGTTAG |
| qPCR-Cu/Zn-SOD-R | GGCCAGAGACATTTCCAGTAA |
| qPCR-MnSOD-F | CGGCAATTAGCGGTGACATA |
| qPCR-MnSOD-R | ATGGCGTCATGTAGCTGTTC |
| qPCR-Ltp1-F | CGAATTTGGCACCTTGTCTTG |
| qPCR-Ltp1-R | GCAGAATTCACCAGAGCCTTA |
| qPCR-ERD10B-F | CAAGGCGGAAGAAGGAAGAA |
| qPCR-ERD10B-R | CGTAGTTGTTGCAGTTGAATGAG |
| qPCR-ACR11-F | CCGGAATCTAGCTCCATGTTAG |
| qPCR-ACR11-R | GACATGGATATGGGTCGCTATG |
| qPCR-Gln1-5-F | GAGAGAGAAGGGAAGGGATACT |
| qPCR-Gln1-5-R | CTCGGATAGGATAGTGGTCTCA |
| qPCR-GLU-F | GGCATGTCTCTTGGAGCTATT |
| qPCR-GLU-R | CACCTTCTCCTGAGTTCGATTT |

**Table S2** Basic information on the 11 AsGSs identified in this study.

| **Gene name** | **Sequence ID** | **Chromosome** | **Coordinate (5′-3′)** | **Protein** | | | **Subcellular localization** |
| --- | --- | --- | --- | --- | --- | --- | --- |
|  |  |  |  | **Length (aa)** | **pI** | **MW(kDa)** |  |
| AsGS1-1-6A | AVESA.00001b.r3.6Ag0000906.1 | 6A | 211776213:211779848 | 378 | 5.73 | 41.37 | Cytoplasmic |
| AsGS1-1-6C | AVESA.00001b.r3.6Cg0002845.5 | 6C | 480809296:480812808 | 326 | 6.11 | 35.82 | Cytoplasmic |
| AsGS1-1-6D | AVESA.00001b.r3.6Dg0000605.1 | 6D | 182133829:182137516 | 356 | 5.39 | 39.04 | Cytoplasmic |
| AsGS1-2-1D | AVESA.00001b.r3.1Dg0003009.1 | 1D | 437279086:437281626 | 382 | 5.52 | 41.77 | Cytoplasmic |
| AsGS1-2-4C | AVESA.00001b.r3.4Cg0003652.1 | 4C | 698148550:698151330 | 385 | 6.03 | 42.31 | Cytoplasmic |
| AsGS1-2-5A | AVESA.00001b.r3.5Ag0000156.1 | 5A | 11201493:11204959 | 383 | 5.46 | 41.72 | Cytoplasmic |
| AsGS1-3-4A | AVESA.00001b.r3.4Ag0001302.1 | 4A | 273719620:273722773 | 364 | 5.52 | 39.87 | Cytoplasmic |
| AsGS1-3-4D | AVESA.00001b.r3.4Dg0001645.1 | 4D | 299399425:299402458 | 411 | 5.97 | 44.78 | Cytoplasmic, Chloroplast |
| AsGS2-2A | AVESA.00001b.r3.2Ag0002305.3 | 2A | 400133772:400137902 | 341 | 6.23 | 37.28 | Extracellular |
| AsGS2-2C | AVESA.00001b.r3.2Cg0003004.1 | 2C | 554835573:554840899 | 431 | 5.76 | 47 | Chloroplast, Cytoplasmic, Mitochondrial |
| AsGS2-2D | AVESA.00001b.r3.2Dg0002227.2 | 2D | 191841806:191845830 | 363 | 8.68 | 39.93 | Mitochondrial, Extracellular |

**Table S3** List of 10 motifs with basic information.

| **Motif** | **E-value** | **Sites** | **Width** |
| --- | --- | --- | --- |
| 1 | 9.8e-420 | 11 | 50 |
| 2 | 1.6e-416 | 11 | 50 |
| 3 | 3.4e-387 | 11 | 50 |
| 4 | 4.9e-315 | 9 | 50 |
| 5 | 5.6E-292 | 9 | 50 |
| 6 | 3.7E-222 | 10 | 41 |
| 7 | 6.1E-189 | 11 | 29 |
| 8 | 1.5E-47 | 3 | 50 |
| 9 | 7.8E-32 | 11 | 8 |
| 10 | 3.2E-27 | 9 | 8 |

**Table S4** The Ka/Ks ratios of AsGS collinear gene pairs in oat.

| **Sequence 1** | | **Sequence 2** | | **Ka** | **Ks** | **Ka/Ks** | **Selection pressure** |
| --- | --- | --- | --- | --- | --- | --- | --- |
| **Name** | **ID** | **Name** | **ID** |  |  |  |  |
| *AsGS1-1-6A* | AVESA.00001b.r3.6Ag0000906.1 | *AsGS1-1-6C* | AVESA.00001b.r3.6Cg0002845.5 | 0.006040301 | 0.125092961 | 0.048286499 | Purifying selection |
| *AsGS1-1-6A* | AVESA.00001b.r3.6Ag0000906.1 | *AsGS1-1-6D* | AVESA.00001b.r3.6Dg0000605.1 | 0.001226994 | 0.016021971 | 0.076581972 | Purifying selection |
| *AsGS1-2-1D* | AVESA.00001b.r3.1Dg0003009.1 | *AsGS1-2-4C* | AVESA.00001b.r3.4Cg0003652.1 | 0.021871316 | 0.125415563 | 0.174390767 | Purifying selection |
| *AsGS1-2-1D* | AVESA.00001b.r3.1Dg0003009.1 | *AsGS1-2-5A* | AVESA.00001b.r3.5Ag0000156.1 | 0.015285244 | 0.075702715 | 0.201911442 | Purifying selection |
| *AsGS1-2-4C* | AVESA.00001b.r3.4Cg0003652.1 | *AsGS1-2-5A* | AVESA.00001b.r3.5Ag0000156.1 | 0.022700346 | 0.136244793 | 0.16661441 | Purifying selection |
| *AsGS1-3-4A* | AVESA.00001b.r3.4Ag0001302.1 | *AsGS1-3-4D* | AVESA.00001b.r3.4Dg0001645.1 | 0.01464354 | 0.129037086 | 0.11348319 | Purifying selection |
